# Integrative exploration of genomic profiles for triple negative breast cancer identifies potential drug targets

**DOI:** 10.1097/MD.0000000000004321

**Published:** 2016-07-29

**Authors:** Xiaosheng Wang, Chittibabu Guda

**Affiliations:** a School of Basic Medicine and Clinic Pharmacy, China Pharmaceutical University, Nanjing, China; b Department of Genetics, Cell Biology and Anatomy; c Bioinformatics and Systems Biology Core; d Department of Biochemistry and Molecular Biology; e Fred and Pamela Buffet Cancer Center, University of Nebraska Medical Center, Omaha, Nebraska, USA.

**Keywords:** copy number variation, gene expression profiling, methylation, microRNA, somatic mutation, targeted therapy, triple negative breast cancer

## Abstract

**Background:**

Triple negative breast cancer (TNBC) is high-risk due to its rapid drug resistance and recurrence, metastasis, and lack of targeted therapy. So far, no molecularly targeted therapeutic agents have been clinically approved for TNBC. It is imperative that we discover new targets for TNBC therapy.

**Objectives:**

A large volume of cancer genomics data are emerging and advancing breast cancer research. We may integrate different types of TNBC genomic data to discover molecular targets for TNBC therapy.

**Data sources:**

We used publicly available TNBC tumor tissue genomic data in the Cancer Genome Atlas database in this study.

**Methods:**

We integratively explored genomic profiles (gene expression, copy number, methylation, microRNA [miRNA], and gene mutation) in TNBC and identified hyperactivated genes that have higher expression, more copy numbers, lower methylation level, or are targets of miRNAs with lower expression in TNBC than in normal samples. We ranked the hyperactivated genes into different levels based on all the genomic evidence and performed functional analyses of the sets of genes identified. More importantly, we proposed potential molecular targets for TNBC therapy based on the hyperactivated genes.

**Results:**

Some of the genes we identified such as *FGFR2*, *MAPK13*, *TP53*, SRC family, MUC family, and BCL2 family have been suggested to be potential targets for TNBC treatment. Others such as CSF1R, EPHB3, TRIB1, and LAD1 could be promising new targets for TNBC treatment. By utilizing this integrative analysis of genomic profiles for TNBC, we hypothesized that some of the targeted treatment strategies for TNBC currently in development are more likely to be promising, such as poly (ADP-ribose) polymerase inhibitors, while the others are more likely to be discouraging, such as angiogenesis inhibitors.

**Limitations:**

The findings in this study need to be experimentally validated in the future.

**Conclusion:**

This is a systematic study that combined 5 different types of genomic data to molecularly characterize TNBC and identify potential targets for TNBC therapy. The integrative analysis of genomic profiles for TNBC could assist in identifying potential new therapeutic targets and predicting the effectiveness of a targeted treatment strategy for TNBC therapy.

## Abstract 2

Supplemental Digital Content is available in the text

## 1 Introduction

Approximately 10% to 20% of breast cancers are triple negative breast cancer (TNBC), a breast tumor subtype that is clinically negative for expression of the estrogen receptor (ER) and progesterone receptor (PR) and lacks overexpression of the human epidermal growth factor receptor 2 (HER2).^[1]^ TNBC often has a poor prognosis due to its aggressive clinical behavior and lack of response to hormonal therapy or therapies that target HER2 receptors. So far, chemotherapy remains the only possible therapeutic strategy in the adjuvant or metastatic setting in TNBC.^[2]^ For example, a latest neoadjuvant trial has shown that the addition of carboplatin to a standard neoadjuvant chemotherapy regimen significantly increased the pathologic complete response in TNBC patients.^[3]^

Some potential targeted-therapy-based approaches to TNBC treatment have been investigated such as targeting vascular endothelial growth factor (VEGF), epidermal growth factor receptor (EGFR), mammalian target of rapamycin (mTOR), and poly (ADP-ribose) polymerase 1 (PARP1).^[4]^ One encouraging result from clinical trials has shown that the PARP inhibitor, Veliparib, can improve pathologic complete response for TNBC patients by combined addition of carboplatin to standard presurgery chemotherapy.^[5]^ However, clinical efficacies for most of targeted therapy remain unclear so far. Thus, discovery of new treatment targets and strategies for TNBC therapy is pressing and of significant interest.

A large volume of cancer genomics data are emerging and advancing breast cancer research.^[6,7]^ The Cancer Genome Atlas (TCGA) Network gave comprehensive molecular portraits of human breast cancer by integrating various types of “omics” data including genomic DNA copy number arrays,^[8]^ DNA methylation, exome sequencing, messenger RNA arrays, microRNA (miRNA) sequencing, and reverse-phase protein arrays. The related investigations have greatly advanced our understanding of breast cancer in molecular profiles, although translation of genomic findings into clinical applications remains challenging. The high-quality TCGA primary breast tumor samples and their comprehensive molecular profiles could be an invaluable source of information for molecular exploration of TNBC and discovery of new treatment targets.

In cancer research, gene expression measure is of great importance as it reflects gene activity directly and has successfully been used to stratify cancer into different subtypes.^[9]^ Lehmann et al^[10]^ identified 6 TNBC subtypes based on gene expression profiling and revealed that each subtype was related to unique gene ontologies and pathways. For example, the immunomodulatory subtype was enriched in immune cell processes and signal transduction pathways, while the luminal androgen receptor (LAR) subtype was enriched in androgen receptor signaling pathways. Further, they found that the different subtypes were uniquely sensitive to different agents. For example, the LAR cell lines were uniquely sensitive to bicalutamide (an androgen receptor [AR] antagonist), and the basal-like cell lines preferentially responded to cisplatin.

One major limitation of gene expression analysis is its variability and unsteadiness, as a single measure often leads to misinterpretation. To overcome the limitation, it is crucial to collect other genomic evidence such as DNA copy number alteration (CNA), DNA methylation, miRNA gene expression, and gene mutation data that also reflect gene activity and could cause gene expression change. Although previous studies have associated cancer with genomic changes in copy number, methylation, miRNAs, and gene mutations,^[8,11]^ integration of the different types of genomic data into cancer research remains challenging, but promising. Some previous studies have used integrative approaches to analysis of TNBC. The following study of Lehmann et al^[10]^ revealed that PIK3CA kinase domain mutations were frequent in the LAR subtype, and the combination of AR antagonism and PI3K inhibition could synergistically inhibit LAR TNBC cell growth.^[12]^ This study exemplifies the importance of integrating different types of genomic data into exploration of discovery of cancer treatment targets. Shah et al^[13]^ revealed that TNBCs exhibit a wide and continuous spectrum of genomic evolution by analyzing somatic mutation, CNA, gene fusions, and gene expression patterns of 104 primary TNBCs. Craig et al^[14]^ integrated gene expression and somatic mutation profiling of 14 metastatic TNBCs using next-generation sequencing data and proposed potential therapeutic targets in metastatic TNBC.

Although these integrative analyses have provided important insights into TNBC,^[12–14]^ a broader exploration of genomic profiles for TNBC could improve our understanding of this disease and detect potential targets for TNBC treatment. In this study, we carried out an integrative exploration of wide genomic profiles (gene expression, copy number, DNA methylation, miRNA gene expression, and gene mutation) for TNBC using the TCGA breast cancer data. In addition to dissecting the biology of TNBC, we attempt to find genes or pathways that could be targets for treatment of TNBC by identification of abnormally hyperactivated genes and pathways in TNBC. Here, we defined the abnormally hyperactivated genes as those genes that have higher expression, more copy numbers, lower methylation level, or are targets of miRNAs with lower expression in TNBC than in normal samples. Based on the different genomic evidences, we categorized the abnormally hyperactivated genes into different levels. The greater the indication that a gene is hyperactivated, the higher the level the gene belongs to. The genes in high levels are more likely to be associated with the pathogenesis of TNBC and therefore could be preferential targets for TNBC treatment.

## 2 Methods

### 2.1 Datasets

We downloaded the breast carcinoma gene expression (microarray), copy number, methylation, miRNA (Level 3), and gene somatic mutation data (Level 2) from the TCGA data portal (https://tcga-data.nci.nih.gov/tcga/dataAccessMatrix.htm). In the gene expression data, we found a total of 55 TNBC samples. Considering that the gene expression activity is our primary concern, and for statistical consistency, we analyzed the same 55 TNBC samples in the other 4 data types. There are 2, 0, 2, and 1 samples missing in copy number, methylation, miRNA, and gene somatic mutation data, respectively. Thus, we analyzed 55 TNBC samples in the gene expression and methylation data, 53 TNBC samples in the copy number and miRNA data, and 54 TNBC samples in the gene mutation data. Ethical approval was waived since we used only publicly available data and materials in this study.

### 2.2 Identification of genes with differential expression, copy number, or methylation level between TNBC and normal samples

Based on the microarray gene expression data, we identified significantly upregulated genes in the TNBC samples, relative to the paired normal samples with at least two-fold mean expression difference (Wilcox signed-rank test, FDR [false discovery rate] ≤0.05).

For the CNA, we used the “∗.nocnv_hg19.seg.txt” data (SNP array 6.0). We annotated the overlapping genes with the genomic regions in the data using the tool PennCNV^[15]^ and obtained the gene copy number by averaging the segment values of the same gene. We identified the genes having significant copy number gain in the TNBC samples relative to the paired normal samples with at least 1.2-fold mean copy number difference (Wilcox signed-rank test, FDR ≤ 0.05). Because the copy number difference was generally low with the maximum being 1.5, we used the 1.2-fold threshold to define the genes with significant copy number difference between the TNBC and normal samples.

In the methylation analysis, we used the data produced by 2 different platforms: HumanMethylation27 (HM27) BeadChip and HumanMethylation450 (HM450) BeadChip. The HM27 data include 32 TNBC samples versus 27 normal samples, and the HM450 data includes 23 TNBC samples versus 47 normal samples. We identified the hypomethylated genes in the TNBC samples relative to normal samples with mean methylation level (β value) difference no less than 5% (Wilcox sum-rank test, FDR ≤ 0.05) in both datasets and selected the genes overlapping between both analyses as the hypomethylated genes in TNBC.

The FDR was estimated using the method of Benjami and Hochberg.^[16]^

### 2.3 Identification of genes that are targets of miRNAs with differential expression between TNBC and normal samples

We identified significantly downregulated *miRNA* genes in the 53 TNBC samples relative to 103 normal samples with at least two-fold mean expression difference (*t* test, FDR ≤ 0.05). Using the tool TargetScanHuman for predicting miRNA targets,^[17]^ we identified the genes that are targets of the downregulated *miRNA* genes. As previously, the FDR was estimated using the method of Benjami and Hochberg.^[16]^

### 2.4 Identification of genes frequently mutated in TNBC

In the gene somatic mutation analysis, we used the MAF (mutation annotation format) data by exome-sequencing data analysis. We first constructed an *m* × *n* mutation matrix, where *m* is the number of genes and *n* is the number of breast cancer samples in the MAF data. The entry (*i*, *j*) in the matrix is 1 if at least 1 mutation in gene *i* was detected in sample *j*, otherwise 0. Based on the mutation matrix, we identified some frequently mutated genes in TNBC (Fisher exact test, *P* value <0.05) and compared their mutation rates in TBNC with those in general breast cancer (992 samples). For convenience, in some cases hereafter, we also call the frequently mutated genes abnormally hyperactivated in TNBC, although a gene mutation does not necessarily result to the hyperactivation of the gene.

### 2.5 Evaluation of significance of hyperactivated genes in TNBC

We categorized the identified genes into different levels based on all the genomic evidence. Level 1 includes those genes with significantly higher expression in TNBC samples than in normal samples; Level 2 includes those genes that belong to Level 1 and were identified as abnormally hyperactivated in at least one of the other genomic analyses (copy number, methylation, miRNA, and gene mutation); Level 3 includes those genes that belong to Level 1 and were identified as abnormally hyperactivated in at least two of the other genomic analyses; Level 4 includes those genes that belong to Level 1 and were identified as abnormally hyperactivated in at least three of the other genomic analyses; Level 5 includes those genes that belong to Level 1 and were identified as abnormally hyperactivated in all the other genomic analyses. The higher the level a gene belongs to, the more likely the gene is to be hyperactivated in TNBC.

### 2.6 Functional analysis of the gene sets identified

Using the gene set enrichment analysis (GSEA) software, we classified the hyperactivated genes into different gene families and identified the gene sets that are significantly overlapping with them. We inferred significant networks associated with gene sets using Ingenuity Pathway Analysis tool (IPA, Ingenuity® Systems, www.ingenuity.com). IPA is a system that yields a set of networks relevant to a list of genes based on the preserved records contained in the Ingenuity Pathways Knowledge Base. We identified significant gene ontology (GO) biological processes that are associated with gene sets using the PANTHER classification system.^[18]^

## 3 Results and discussion

### 3.1 Identification of the abnormally hyperactivated genes in TNBC

We identified 1800 upregulated genes in the TNBC samples with at least 2-fold higher mean expression compared to the normal samples (Wilcox signed-rank test, FDR ≤ 0.05). We identified 1655 genes that have at least 1.2-fold mean copy number gain in the TNBC samples compared to the normal samples (Wilcox signed-rank test, FDR ≤ 0.05). We identified 731 genes that have lower methylation level (β value) in TNBC samples compared to normal samples in both the HM27 and HM450 data analysis with mean β value difference no less than 5% (Wilcox sum-rank test, FDR ≤ 0.05). We identified 2020 *mRNA* genes that are targets of the 52 downregulated miRNAs in the TNBC samples compared to normal samples with at least 2-fold mean expression difference (*t* test, FDR ≤ 0.05). We also identified 18 genes that are frequently mutated in the TNBC samples (Fisher exact test, *P* value <0.05). Here, we refer to the groups of genes identified by gene expression, copy number, methylation, miRNA, and gene mutation analyses as GE, CN, ME, MR, and GM, respectively. These gene lists are shown in the Additional File 1, Table S1.

**Figure 1:**
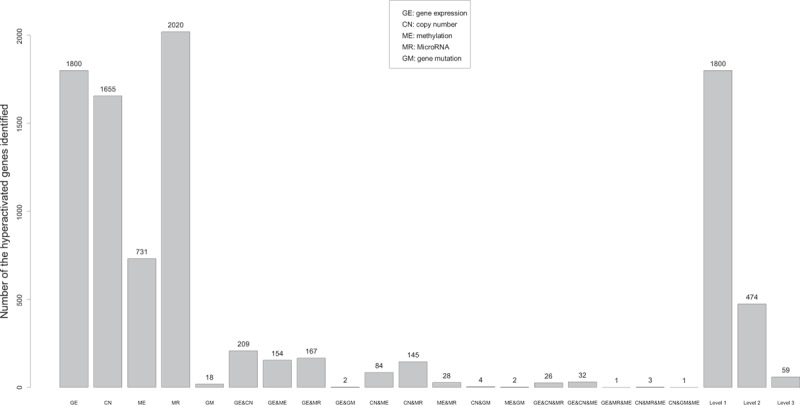
Numbers of the genes identified in all the genomic analyses.

**Figure 2:**
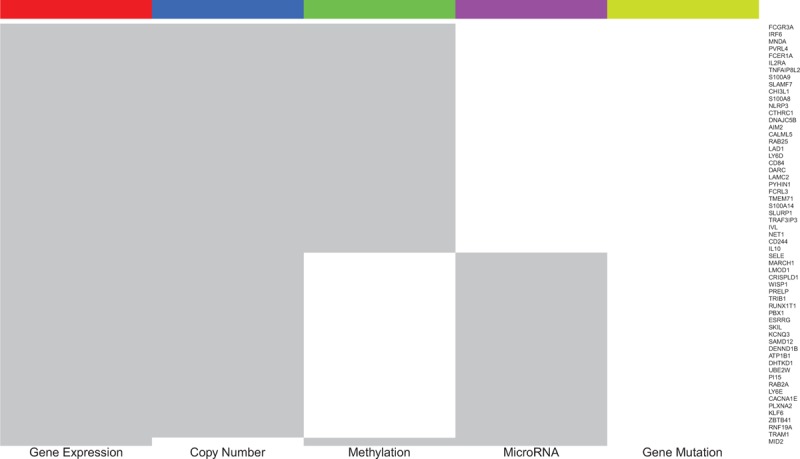
The Level 3 genes and their hyperactivated status in the different genomic analyses. The gray color indicates that the gene is hyperactivated in the analysis, and the white indicates that the gene isn’t hyperactivated in the analysis.

Figure 1 illustrates overlaps between the gene sets identified in the different genomic analyses (also see the Additional File 2, Table S2). For example, there are 209, 154, 167, and 2 genes overlapping between GE and CN, ME, MR, and GM, respectively; there are 32 genes overlapping among GE, CN, and ME. We categorized the identified genes into different levels based on all the genomic evidence. Level 1 includes the 1800 genes that were highly expressed in TNBC samples compared to normal samples; Level 2 includes 474 genes that lie in Level 1 and were hyperactivated in TNBC by at least one of the other genomic analyses; Level 3 includes 59 genes that lie in Level 1 and were hyperactivated in TNBC by at least two of the other genomic analyses. Both Levels 4 and 5 contain 0 genes. We only explored the genes in Levels 1, 2, and 3 (see the Additional File 1, Table S1). Figure 2 is a heatmap that presents the Level 3 genes and their hyperactivated status in the different genomic analyses.

### 3.2 Functional analysis of the abnormally hyperactivated genes in TNBC

We are more interested in those genes in Levels 2 and 3 because they were not only highly expressed in TNBC but also identified abnormally hyperactivated by other genomic evidence. We classified the Level 2 genes into different gene families using the GSEA software as shown in Table 1.^[19]^

**Table 1:**
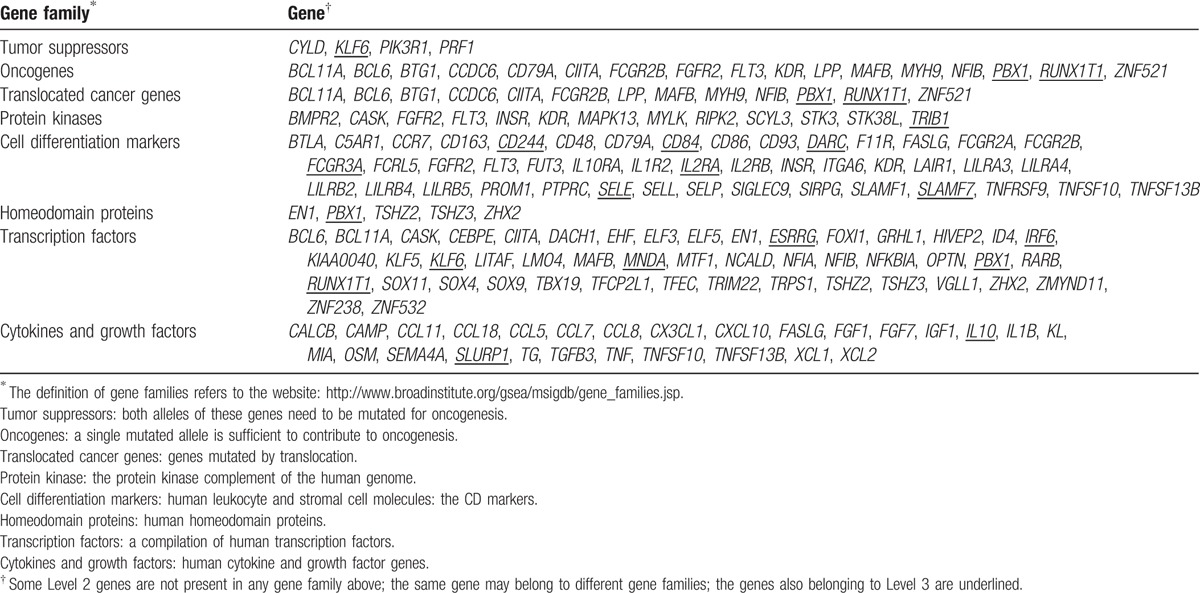
Category of the Level 2 genes.

We used the “Compute Overlaps” tool in GSEA to get the gene sets (positional, curated, or oncogenic) that were significantly overlapping with the Level 2 gene list (FDR < 10^−10^). Among them, a number of gene sets (Table 2) are related to breast cancer, other cancer types, and stem cells. Table 2 shows that the hyperactivated genes we identified in TNBC tend to be upregulated in the basal subtype of breast cancer, breast cancer resistant to chemotherapy, ER breast cancer, and aggressive prostate cancer, lymphoma, acute myeloid leukemia (AML), and hepatocellular carcinoma, underlying the molecular commonalities between TNBC and aggressive cancer types or subtypes. Many of the hyperactivated genes are also highly expressed in stem cells, indicating that the TNBC cells may harbor a substantial number of cancer stem cells that result in invasive activities of TNBC. In addition, Table 2 shows that many of the hyperactivated genes in TNBC are associated with dysregulation of TP53, aberrant activation of the Wnt signaling pathway, and immune system processes. These features have been correlated with aggressiveness and poor prognosis of breast cancers.^[20–22]^

**Table 2:**
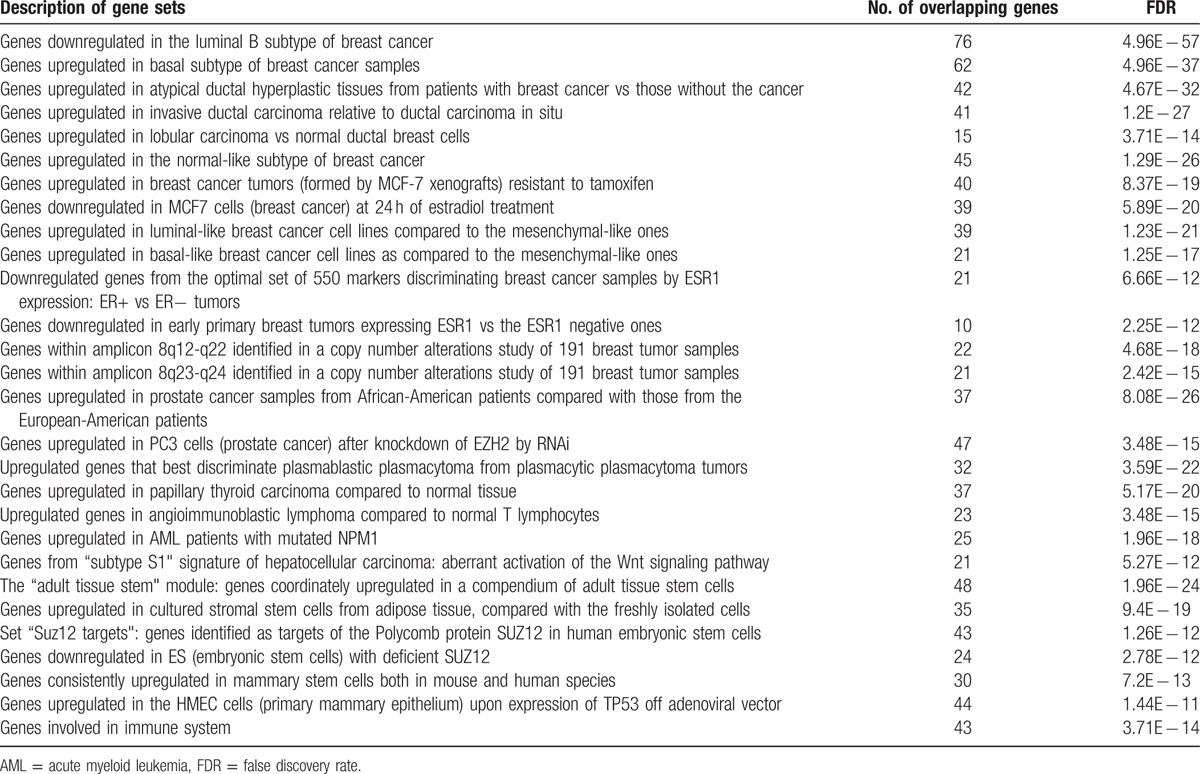
Gene sets that are significantly overlapping with the Level 2 gene list.

**Figure 3:**
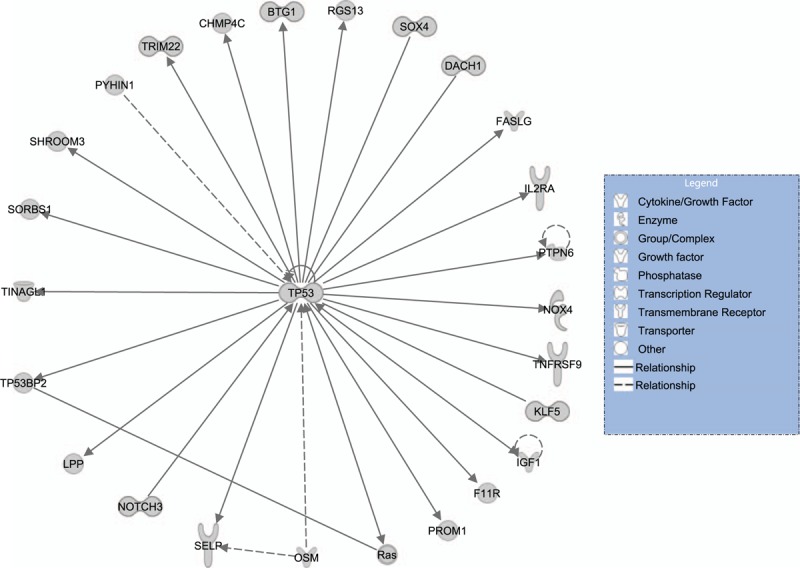
TP53-centered protein–protein interaction network identified based on the Level 2 gene set using Ingenuity Pathway Analysis.

We performed a network analysis of the Level 2 gene set with the addition of the tumor suppressor gene *TP53*, since dysregulation of TP53 has been found in the vast majority of TNBC cases.^[23]^ In our analyses, TP53 mutation was highly frequent (78% mutation rate) in TNBC, and its expression was significantly lower in TNBC than in normal samples (1.6-fold mean expression difference, Wilcox signed-rank test, FDR = 0.002). We generated a TP53-centered network (Fig. 3), in which TP53 connects to all the other nodes. Figure 3 shows that TP53 regulates many hyperactivated genes such as *RGS13*, *SOX4*, *NOTCH3*, *TRIM22*, and *IGF1*, and genes associated with RAS signaling. Dysregulation of TP53 may be associated with abnormal hyperactivation of these regulated genes and pathways that significantly contribute to pathogenesis and progression of TNBC.

### 3.3 Identification of genes that are frequently mutated in TNBC

**Figure 4:**
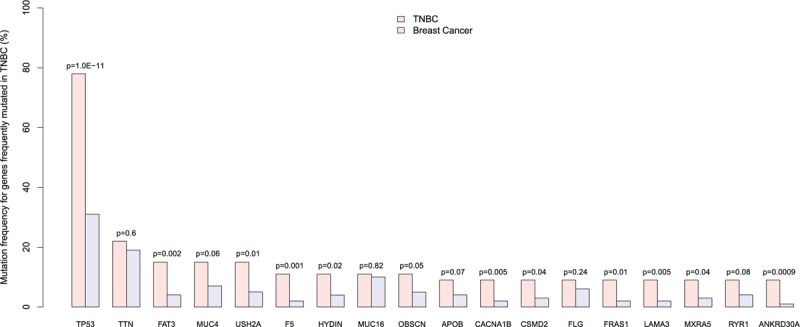
Compare mutation frequency of the frequently-mutated genes in between TNBC and breast cancer in general. The Fisher exact test *P* values are presented.

In the 54 TNBC samples with exome-sequencing data, we found 18 genes that were frequently mutated (Fisher exact test, *P* value <0.05) as shown in Table 3. Notably, TP53 has the highest mutation rate (78%) that is much higher than its 31% mutation rate across all the TCGA breast cancers (odds ratio: 7.7, Fisher exact test *P* value = 10^−11^), suggesting that TP53 mutations might significantly contribute to aggressiveness of TNBC. TTN has the second highest mutation rate (22%) in TNBC, slightly higher than its 19% mutation rate across all the breast cancers. Table 3 and Fig. 4 show that a majority of the frequently mutated genes in TNBC have significantly higher mutation frequency compared to breast cancer in general, suggesting that mutations in these genes may contribute to higher aggressiveness of TNBC compared to non-TNBC breast cancers.

**Table 3:**
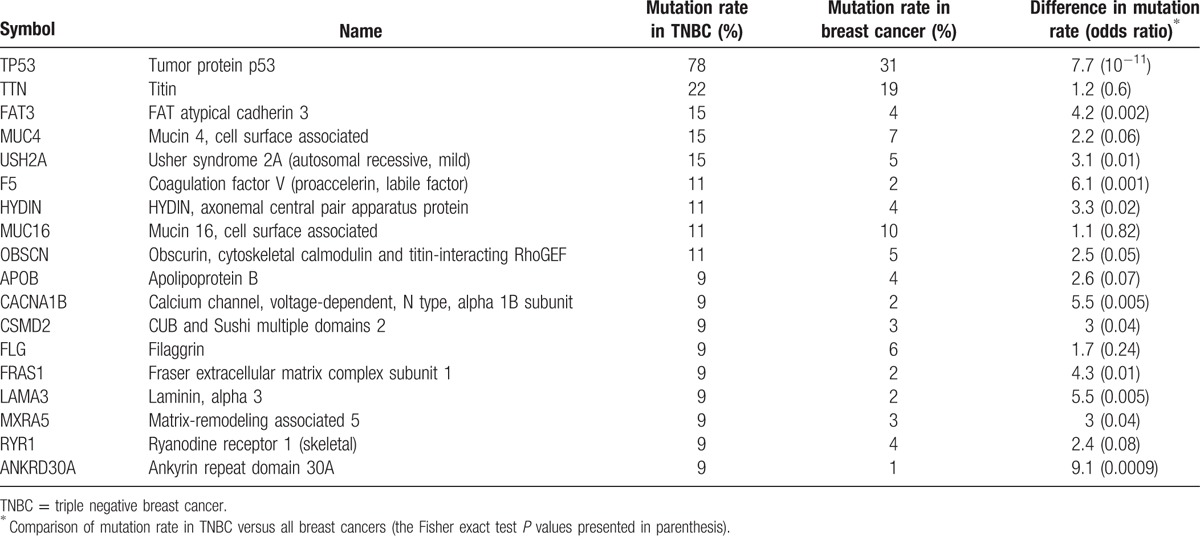
Genes frequently mutated in TNBC.

Using the PANTHER classification system,^[18]^ we identified significant GO biological processes associated with the 18 frequently mutated genes as shown in Table 4. Table 4 shows that these genes are mostly involved in important biological processes that underlie the pathogenesis of cancer.

**Table 4:**
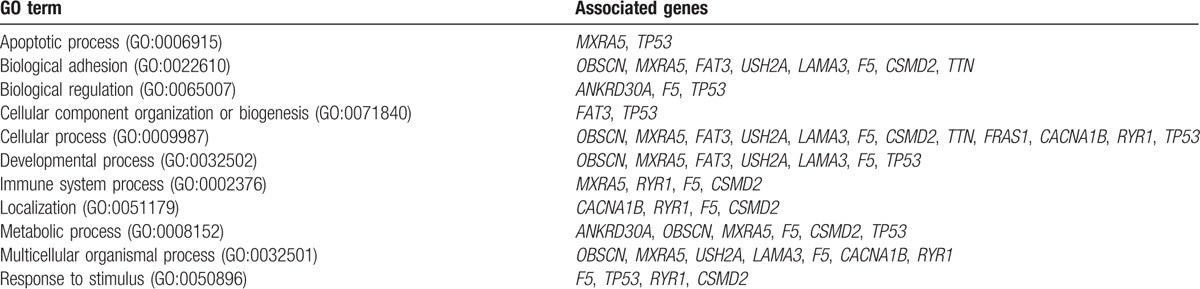
Gene ontology related to the highly mutated genes in TNBC.

In Table 3, 2 members of the MUC gene family, *MUC4* and *MUC16*, show high frequency of mutation in TNBC. It has been shown that MUC4 could promote invasive activities of TNBC and be associated with metastasis of breast cancer^[24,25]^ and MUC16 could increase proliferation and antiapoptosis in breast cancer cells,^[26]^ consistent with their high mutation rate in the aggressive TNBC. Interestingly, both MUC4 and MUC16 had decreased expression in TNBC compared to normal samples (Wilcox signed-rank test, *P* value = 2 × 10^−5^ and 0.035 for MUC4 and MUC16, respectively), but highly expressed in TNBC compared to non-TNBC tumor samples (*t* test, *P* value = 2.2 × 10^−6^ and <10^−7^ for MUC4 and MUC16, respectively). This is similar to a previous finding that MUC4 expression was depressed in primary breast tumors relative to normal tissue, but was elevated in metastatic lesions compared to primary breast tumors,^[24]^ suggesting that MUC4 may play an important role in promoting TNBC metastasis. Except for MUC4 and MUC6, other *MUC* genes also have mutations in TNBC (Table 5). In fact, *MUC* genes have been identified as attractive therapeutic targets since their deregulation has been associated with unfavorable prognosis of cancers.^[27]^

**Table 5:**
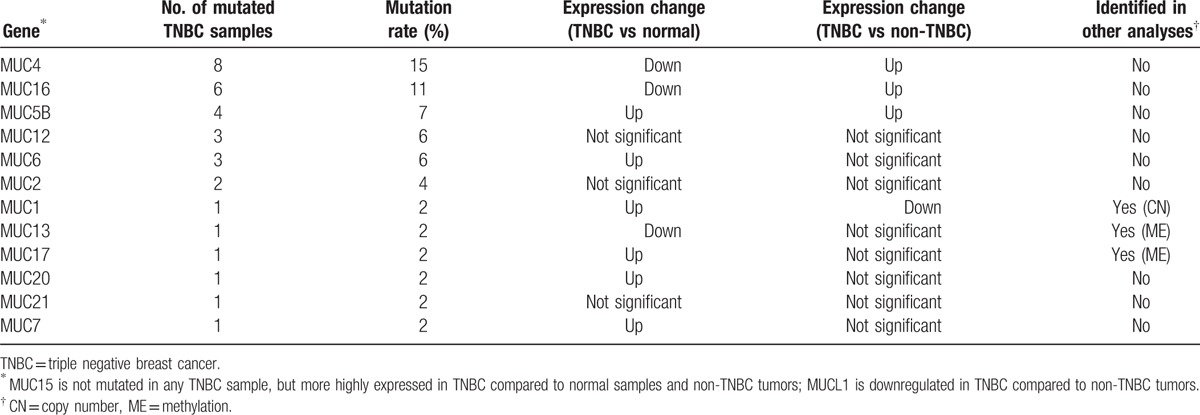
*MUC* genes mutated in TNBC.

### 3.4 Identification of potential targets for TNBC therapy

#### 3.4.1 The hyperactivated kinase-encoding genes could be promising targets for TNBC therapy

It has been recognized that many kinase-encoding genes are upregulated in cancer and the development of anticancer drugs that inhibit overexpression of protein kinases is promising in cancer treatment.^[28,29]^ Therefore, of the hyperactivated genes identified in TNBC, the druggable kinase genes could be good candidates for development of molecularly targeted therapy for TNBC. Table 6 presents the highly expressed kinase genes (Levels 1 and 2) in TNBC compared to normal samples (at least 2-fold expression elevation, Wilcox signed-rank test, FDR < 0.05).

**Table 6:**
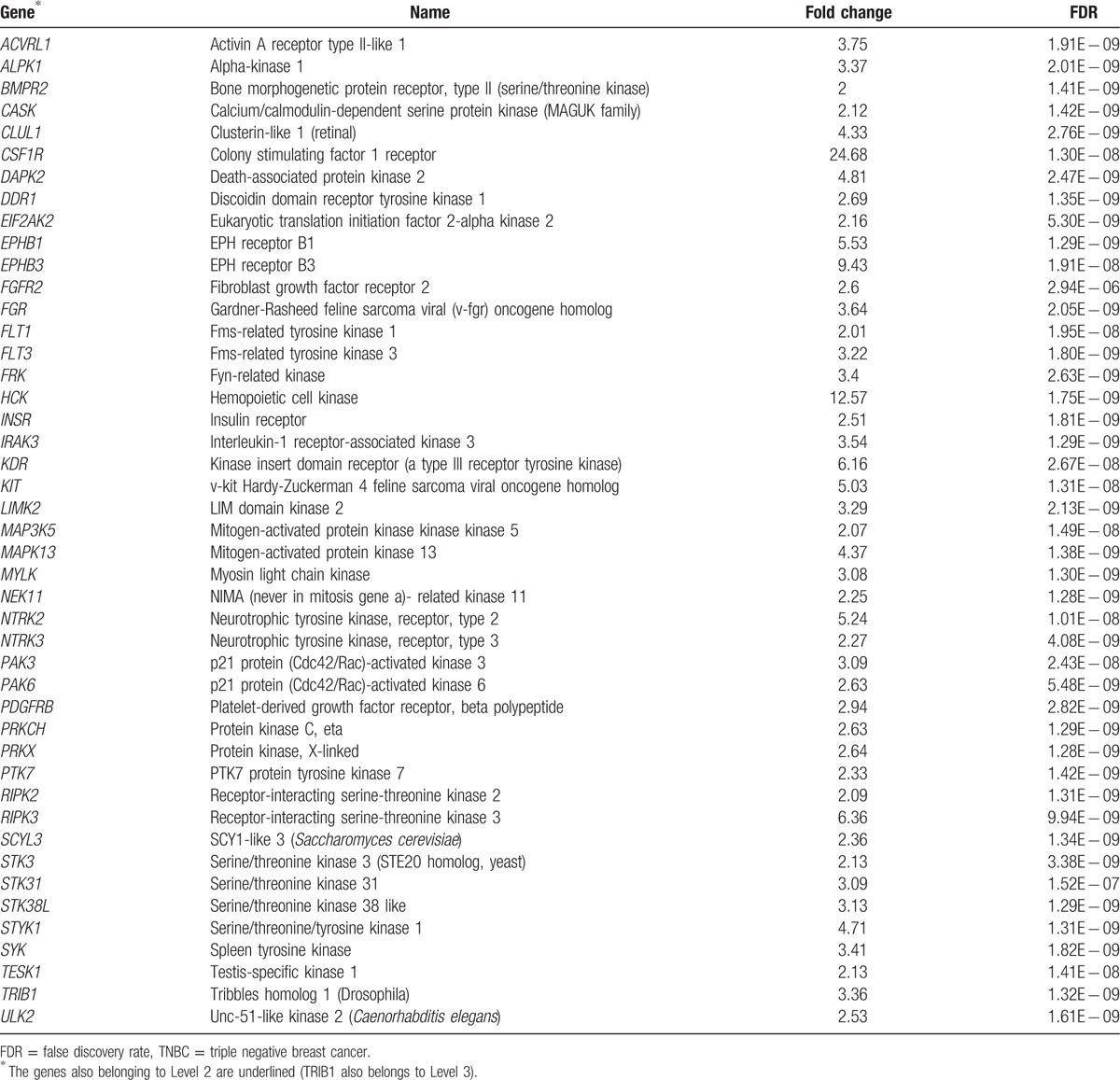
Kinase-encoding genes highly expressed in TNBC.

Of the kinase genes in Table 6, CSF1R has the highest expression elevation in TNBC (24.68-fold expression elevation, FDR = 1.30 × 10^−8^). Previous studies have revealed that overexpression of CSF1R was associated with ipsilateral breast cancer recurrence and poor prognosis of breast cancer.^[30]^ This is in line with our result that CSF1R is highly expressed in TNBC, which often has unfavorable clinical outcome. Therefore, CSF1R could be an important target for TNBC therapy. In fact, it has been shown that CSF1R activity could be inhibited by some small molecule inhibitors such as imatinib, dasatinib, sunitinib, CEP-701, and PKC-412.^[31]^ These compounds may be worth clinical trial for TNBC therapy.

Table 6 presents many kinase genes that belong to the same gene families (including *SRC*, *EPH*, *FLT*, *MAP*, *NTRK*, *PAK*, *PRK*, *RIPK*, and *STK*) that are worth investigation. For example, HCK has the second highest expression elevation in TNBC (12.57-fold expression elevation, FDR = 1.75 × 10^−9^). The gene encodes a member of the SRC family of tyrosine kinases, which are potential therapeutic targets for TNBC.^[32,33]^ In Table 6, there is another SRC family kinase gene *FGR* that are overexpressed (3.64-fold expression elevation, FDR = 2.05 × 10^−9^) and amplified in TNBC (1.5-fold copy number gain, FDR = 1.35 × 10^−8^). Some small molecule inhibitors such as dasatinib have been shown to be effective in TNBC therapy, possibly because they can inhibit the activity of the SRC family kinases.^[34]^ EPHB3, a member of the EPH receptor gene family, has the third highest expression elevation in TNBC (9.43-fold expression elevation, FDR = 1.91 × 10^−8^). Another EPH receptor family gene, *EPHB1*, is also highly expressed in TNBC (5.23-fold expression elevation, FDR = 1.29 × 10^−9^). It has been reported that increased expression of the EPH receptor was correlated with more malignant and metastatic tumors,^[35]^ which is consistent with our results.

The kinase genes in Level 2 are especially worthy of note since their hyperactivation in TNBC was confirmed or demonstrated by multiple genomic evidences. For example, FGFR2 (fibroblast growth factor receptor 2) has more than 2-fold higher expression in TNBC (FDR = 2.94 × 10^−6^) and is targeted by miRNA-410 and miRNA-381, both of which were significantly downregulated in TNBC compared to the normal samples (*t* test, FDR < 10^−6^). This gene has been found to be hyperactive in breast cancer and is associated with increased breast cancer risk.^[36]^ Another study has shown that FGFR2 was amplified in TNBC cell lines that were highly sensitive to FGFR2 inhibitors.^[37]^ MAPK13 (mitogen-activated protein kinase 13) has more than 4-fold higher expression (FDR < 1.38 × 10^−9^) and much lower methylation level in TNBC than in the normal samples (FDR < 3 × 10^−7^). The gene is involved in MAPK pathways that have been suggested to be potential targets for TNBC treatment.^[38]^ TRIB1 has more than 2-fold higher expression in TNBC (FDR = 1.32 × 10^−9^), 1.4-fold copy number gain (FDR = 10^−8^), and is targeted by miRNA-144, which was significantly downregulated in TNBC compared to the normal samples (*t* test, FDR = 5.85 × 10^−11^). This gene plays a role in mediating proliferation, apoptosis, and differentiation in cells through binding to MAPKK signaling proteins of MAPK pathways, and has been suggested as a therapeutic target for prostate cancer.^[39]^ Our results suggest that this gene could be a promising target for TNBC therapy.

In summary, the kinase genes hyperactivated in TNBC provide potential targets for development of molecularly targeted therapy for TNBC.

#### 3.4.2 Identification of the hyperactivated genes that are targets of TNBC-sensitive agents

TNBC is highly concordant with basal-like breast cancer (BLBC), defined by gene expression profiling, in that both share many clinical features such as lack of expression of ER, PR, and HER2, high p53 mutation rate, unfavorable clinical outcome, and so on.^[2,8]^ In addition, the majority of claudin-low tumors are triple negative and with poor prognosis.^[40]^ In a previous study, Heiser et al^[41]^ revealed that different breast cancer subtypes (luminal, basal, HER2-enriched, and claudin-low) exhibited differential sensitivities to most therapeutic compounds by performing a systematic drug screening of breast cancer cell lines. They identified a list of compounds that showed significant subtype specificity (Table 1 of Ref. ^[41]^), in which we found that three of the seven basal-like and claudin-low subtype sensitive compounds target genes in the list of hyperactivated genes we identified. The three compounds include docetaxel, PD173074, and CGC-11047, which could be promising in molecularly targeted TNBC therapy (Table 7). In fact, docetaxel has been reported to be effective in TNBC treatment^[42]^; PD173074 has been shown to be able to impair breast cancer metastasis by inhibiting FGFR signaling^[43]^; CGC-11047 has been suggested to be preferentially effective against aggressive breast cancer subtypes.^[44]^

**Table 7:**
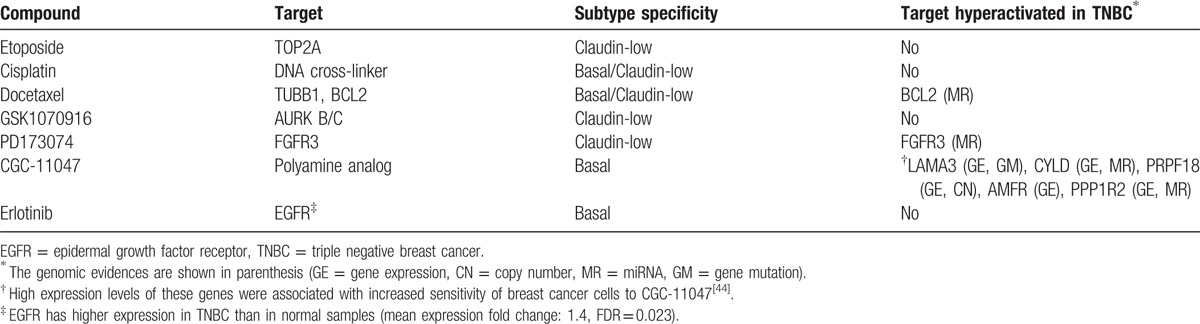
Compounds that are potentially effective in TNBC therapy.

In Table 7, BCL2 is the target of docetaxel that has been used in the neoadjuvant treatment for TNBC.^[45]^ We identified several other *BCL2* family genes that were hyperactivated in TNBC including *BCL2L2* (MR), *BCL2L10* (GE), *BCL2L11* (MR), *BCL2L14* (GE and ME), and *MCL1* (CN). In addition, BCL2L11 and BCL2L12 were found to be mutated in 1 sample, and BCL2A1 has higher expression in TNBC compared to normal samples (mean expression 1.4-fold change, FDR = 0.048). Our results are consistent with previous findings that alterations in *BCL2* family genes were associated with pathogenesis and progression of human cancers.^[46–48]^ Thus, *BCL2* family genes could provide targets for cancer therapy including TNBC.

In another study, Shiang et al^[49]^ identified 224 genes that critically sustain the viability of TBNC cell lines by siRNA screening (Appendix Table A2 in Ref. ^[49]^). Of them, 1 (LAD1), 20, and 58 genes were presented in our Levels 3, 2, and 1 gene list, respectively (Additional File 3, Table S3). The Level 3 gene *LAD1* encodes a protein that may contribute to the stability of the association of the epithelial layers with the underlying mesenchyme. Its role in TNBC is unappreciated, but worth further investigation, since the gene was highly expressed (4-fold expression elevation, FDR = 5.93 × 10^−9^), amplified (1.34-fold copy number gain, FDR = 1.03 × 10^−7^), and lower-methylated (β value depression >5%, FDR < 2 × 10^−5^) in TNBC compared to normal samples.

#### 3.4.3 Genomic profiles for targets of the agents currently being explored in clinical trials

Currently, there are several targeted agents in development for the treatment of metastatic TNBC.^[4]^ The targets of these agents include VEGF, EGFR, PARP, mTOR, FGFR, JAK2, AR, NOTCH, HDAC, and MET (Table 3 of Ref. ^[4]^). We examined the genomic profiles for these genes in TNBC as shown in Table 8. It can be seen from Table 8 that some of the genes (families) are generally upregulated in TNBC such as EGFR, PARP family, and NOTCH family, while some others are downregulated in TNBC such as VEGF family. It could partially explain that in experimental and clinical trials to test new treatment for TNBC, the agents targeting EGFR and PARP family have shown encouraging results,^[50,51]^ while the agents targeting VEGF showed conflicting results.^[45]^Table 8 indicates that the FGFR family member FGFR2 could be a good therapeutic target for TNBC relative to the other FGFR family members. The *NOTCH* family genes are consistently upregulated in TNBC, indicating that NOTCH inhibition could be effective in TNBC therapy. In the HDAC family, some genes are hyperactivated in TNBC such as *HDAC2*, *HDAC5*, *HDAC6*, *HDAC9*, and *HDAC11*. Inhibition of them could be promising in TNBC therapy. For the targeted treatments against mTOR, JAK2, AR, or MET, Table 8 shows no strong evidence supporting that they could be effective in TNBC therapy. Certainly, the association between genomic profiles and efficacy of the targeted therapy needs to be confirmed by more clinical experiments with genomic data available.

**Table 8:**
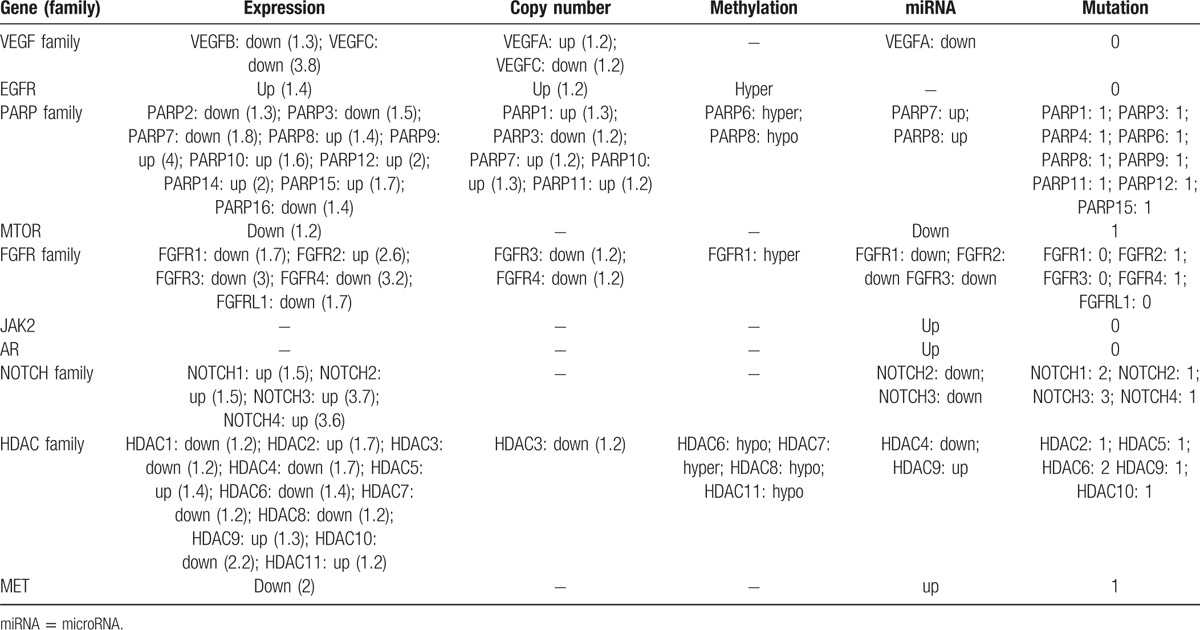
Genomic profiles for targets of the agents currently explored in clinical trials.

## 4 Conclusion

TNBC is high-risk due to its rapid drug resistance and recurrence, metastasis, and lack of targeted therapy. So far, no molecularly targeted therapeutic agents have been clinically approved for TNBC. Treatments that target molecules such as EGFR, VEGF, PARP, and mTOR are still at an early stage of research. It is essential for us to discover new treatment targets for TNBC. The cancer genomics data are becoming an invaluable source for development of molecular targets for TNBC therapy.^[8]^ In the present study, we integrally explore genomic profiles (gene expression, copy number, methylation, miRNA, and gene mutation) in TNBC. To our knowledge, this is the first study that combined the 5 different types of genomic data to molecularly characterize TNBC and identify potential targets for TNBC therapy. We identified hyperactivated genes in TNBC based on multiple genomic evidences, which could significantly contribute to pathogenesis and progression of TNBC. Our results confirm previous findings that TNBC has common molecular profiles with BLBC subtype. Moreover, we revealed that many of the hyperactivated genes in TNBC were also highly active in invasive cancer types or subtypes such as lymphoma, AML, hepatocellular carcinoma and invasive prostate cancer, and stem cells, suggesting that their high activities may contribute to the aggressiveness of cancer.

In the present study, we identified potential molecular targets for TNBC therapy. Some of them such as FGFR2, MAPK13, TP53, SRC family, MUC family, and BCL2 family have been suggested to be potential targets for TNBC treatment by previous studies.^[23,27,33,36,38]^ The others such as CSF1R, EPHB3, TRIB1, and LAD1 could be promising new targets for TNBC treatment for which further investigation is worth doing, whereas their importance in TNBC has not been recognized.

Targeted treatment strategies for TNBC have been developed, some of which were encouraging while others were discouraging.^[45]^ Integrative genomic profiles for TNBC could assist in predicting the effectiveness of a targeted treatment strategy and identifying potential new targets.

In the present study, we treated all the TNBC samples as a single homogeneous group instead of dividing them into several heterogeneous subgroups as shown in Ref. ^[10]^. As a result, the hyperactivated genes we identified could show varied “hyperactivity” across the different subgroups. Dissection of TNBC into different subtypes and discovery of subtype-specific molecular targets for TNBC therapy could be a promising direction for us to make efforts in the future. In addition, based on the same method, using the TCGA and other comprehensive cancer genomic data, we can explore other cancer types to find potential molecular targets for their treatment.

## Supplementary Material

Supplemental Digital Content

## Supplementary Material

Supplemental Digital Content

## Supplementary Material

Supplemental Digital Content
