# Integrative exploration of genomic profiles for triple negative breast cancer identifies potential drug targets: Erratum

**DOI:** 10.1097/01.md.0000503499.57465.64

**Published:** 2016-10-07

In the article “Integrative exploration of genomic profiles for triple negative breast cancer identifies potential drug targets”,^[1]^ which appeared in Volume 95, Issue 30 of *Medicine*, Tables 6 and 8 originally contained errors. The article has since been corrected online.
